# Tree-based classification system incorporating the HVTT-PVTT score for personalized management of hepatocellular carcinoma patients with macroscopic vascular invasion

**DOI:** 10.18632/aging.102403

**Published:** 2019-11-03

**Authors:** Fei Cao, Lujun Shen, Han Qi, Lin Xie, Ze Song, Shuanggang Chen, Weijun Fan

**Affiliations:** 1 Department of Minimally Invasive Interventional Therapy, Sun Yat-sen University Cancer Center, State Key Laboratory of Oncology in South China, Collaborative Innovation Center for Cancer Medicine, Guangzhou 510060, Guangdong, China; 2 Department of Oncology, The Seventh Affiliated Hospital, Sun Yat-sen University, Shenzhen 518107, China

**Keywords:** classification and regression trees, vascular invasion, hepatocellular carcinoma, classification system

## Abstract

Purpose: To develop a decision tree algorithm-based classification system for personalized management of hepatocellular carcinoma (HCC) patients with macroscopic vascular invasion.

Results: The HVTT-PVTT score could differentiate two groups of patients (< 3 and ≥ 3 points) with different survival outcomes (7.4 vs 4.6 months, *P* < 0.001) and surgical proportion (24.4% vs 3.6%, *P* < 0.001). Using the Cox regression model and classification and regression tree (CART) algorithm, patients in the training set were automatically separated into three subgroups with different prognosis (10.3 vs 6.1 vs 3.3 months). The predictive accuracy was verified in the validation group (12.3 vs 6.9 vs 5.6 months) and was better than other commonly used staging systems.

Conclusions: Our study proposed a new classification system for HCC patients with macroscopic vascular invasion that could be meaningful for personalized management of these patients.

Methods: A total of 869 HCC patients initially diagnosed with macroscopic vascular invasion were randomly divided into training and validation sets. A comprehensive and simplified HVTT-PVTT score was set up for subdivision of vascular invasion according to the patients’ survival outcome. Then, a decision tree algorithm-based classification system was used to establish the refined subdivision system incorporating all independent prognostic factors.

## INTRODUCTION

Hepatocellular carcinoma (HCC) is the fifth most common neoplasm and the third leading cause of cancer death worldwide [1]. HCC has a tendency to invade the vascular system, and it has been reported that approximately 40% of HCC patients have a vascular tumor thrombus when first diagnosed [2, 3]. HCC patients with macroscopic vascular invasion have an extremely poor prognosis, with a median overall survival (OS) of 2.7-4.0 months [4, 5]. The Barcelona Clinic for Liver Cancer (BCLC) staging system recommended systemic therapy as the standard therapy [6], but the greatest reported survival benefit of sorafenib monotherapy was less than 3 months [7]. Additionally, HCC with vascular invasion have been gradually accepted to have different disease behaviors and prognosis, and some of these patients may benefit from aggressive treatments [8]. Therefore, sub-classification of HCC with vascular invasion is important for the development of personalized management strategies.

Many sub-classification systems have been developed for HCC with vascular invasion. Previous reports showed that the extent of the portal vein tumor thrombus (PVTT) was associated with the prognosis [9]. In Eastern countries, two classification systems based on the extent of PVTT are widely accepted: Cheng’s classification system (Types I-IV) and the Japanese staging system (Vp1-Vp4) [10, 11]. However, without evaluation of clinical performance status, liver function and tumor burden, these two systems are not comprehensive for management of HCC patients with tumor thrombus [12]. In addition, hepatic vein tumor thrombus (HVTT) including hepatic vein tumor thrombus, inferior vena cava tumor thrombus (IVTT) and right atrium tumor thrombus was not involved in these two staging systems due to their rarity [13, 14]. In fact, the incidence of HVTT has been reported to be 1.4-4.9%, and approximately 10% of HCC cases with PVTT are combined with HVTT [15, 16]. Therefore, HVTT should also be incorporated into the staging system for HCC with vascular invasion. The Hong Kong Liver Cancer (HKLC) staging system was developed containing both HVTT and PVTT and offers more aggressive treatments for HCC patients than BCLC staging system [17]. However, when patients present with intrahepatic tumor thrombus, the prognosis is difficult to predict and the therapeutic strategy seems ambiguous [18]. The HKLC staging system built a totally different classification for HCC patients with tumor thrombus compared with BCLC staging system, but a sub-classification of BCLC-C stage might be more practical because of the widely use of BCLC staging system in clinical.

In this study, to build the classification we used the CART algorithm, which was promoted in 1984 and represents a major milestone in the development of machine learning, data mining, non-parametric statistics, and artificial intelligence. CART is a non-parametric decision tree technique that forms a collection of rules based on variables that can dichotomize populations into subjects using suitable parameters. This algorithm has been highly praised as one of the top ten algorithms in data mining. Recently, it has been gradually incorporated into tumor staging, because it can classify patients into subgroups with different prognosis using selected factors [19–21]. In this study, we propose a decision tree algorithm-based classification system incorporating the HVTT-PVTT score for HCC patients with vascular invasion that may be meaningful for personalized management of these patients.

## RESULTS

### Baseline characteristics

In total, 869 eligible HCC patients with vascular invasion were included in this study. The median age was 49 years (range, 13-80 years). All patients had macroscopic vascular tumor invasion, including PVTT (82%), HVTT (7.1%), or both (10.8%). Approximately one-third (273/869) of the patients had extrahepatic metastases. In terms of the initial therapy reported for the cases included in this study, the first-line treatment strategies included transarterial chemoembolization (TACE) (58.6%), liver resection (17.6%), sorafenib (8.1%), supportive care (14.8%), and radiotherapy (0.9%). During follow-up, 642 (73.9%) patients died. The median OS was 7.0 months (95% confidence interval (CI), 6.37-7.62 months). The median follow-up time was 51.67 months (95 % CI, 44.06-57.11 months). The 869 patients were randomly divided into the training set (426 patients) and validation set (443 patients). The baseline characteristics of the training and validation sets are summarized in Table 1. In summary, the patients in the training and validation sets shared similar characteristics.

**Table 1:** Baseline characteristics of the training and validation sets.

**Baseline characteristics**	**Training set (N=426)**		**Validation set (N=443)**		***P* value**
Median age, y(range)	49	(13-80)	48	(17-78)	0.58
Gender					
Male	395	92.70%	410	92.60%	0.923
Female	31	7.30%	33	7.40%	
ECOG PS 0-1	426	100%	443	100%	
HBsAg					
Positive	400	94%	410	92.60%	0.43
Negative	26	6.10%	33	7.40%	
Laboratory test					
HGB, g/L	133	(65-200)	131	(54-193)	0.203
PLT, ×109/L	170	(30-684)	170	(30-486)	0.773
Child-Pugh					
A	316	74.20%	349	78.80%	0.085
B	110	25.80%	94	21.20%	
AFP level, ng/ml^#^					
≤20	63	14.90%	64	14.50%	0.944
>20 to ≤400	76	17.90%	83	18.80%	
≤400	285	67.20%	294	66.70%	
Number of nodules					
Single	205	48.10%	233	52.60%	0.187
Multiple (≥2)	221	51.90%	210	47.40%	
Maximal tumor size, cm					
≤5	37	8.70%	46	10.40%	0.548
>5 to ≤10	183	43.00%	177	40.00%	
>10	206	48.40%	220	49.60%	
Tumor status					
Low tumor burden	223	52.30%	252	56.90%	0.179
High tumor burden	203	47.70%	191	43.10%	
Extent of portal vein invasion					
None	30	7.00%	32	7.20%	0.667
I(segmental/sectoral)	106	24.90%	115	26.00%	
II (left and/or right main)	187	43.90%	188	42.40%	
III (main trunk)	100	23.50%	100	22.60%	
IV (superior mesenteric vein)	3	0.70%	8	1.80%	
Extent of hepatic vein					
None	341	80%	372	84.00%	0.439
Hepatic vein	53	12.40%	40	9.00%	
Inferior vena cava	27	6.30%	27	6.10%	
Right atrium	5	1.20%	4	0.90%	
Extrahepatic spread					
None	300	70.40%	289	65.20%	0.102
Extrahepatic spread	126	30.60%	154	34.80%	
Main treatment given					
Supportive care	67	15.70%	62	14.00%	0.879
TACE	246	57.70%	263	59.40%	
Radiotherapy	5	1.20%	3	0.70%	
Surgery	75	17.60%	78	17.60%	
Sorafenib	33	7.70%	37	8.40%	
Status at analysis					
Died	318	74.60%	324	73%	0.612
Alive or censored	108	25.40%	119	27%	
Median overall survival, months(range)	6.4	0.07-97.9	7.4	0.07-103.6	0.108

Abbreviations: ECOG PS, Eastern Cooperative Oncology Group performance status; HGB, hemoglobin; PLT, platelets; TACE, trans arterial chemoembolization.

# Data of four patients were missing; * Fisher exact test was used when more than 20% of cells had an expected frequency less than 5.

### Development of HVTT-PVTT scoring system

**Figure 1:**
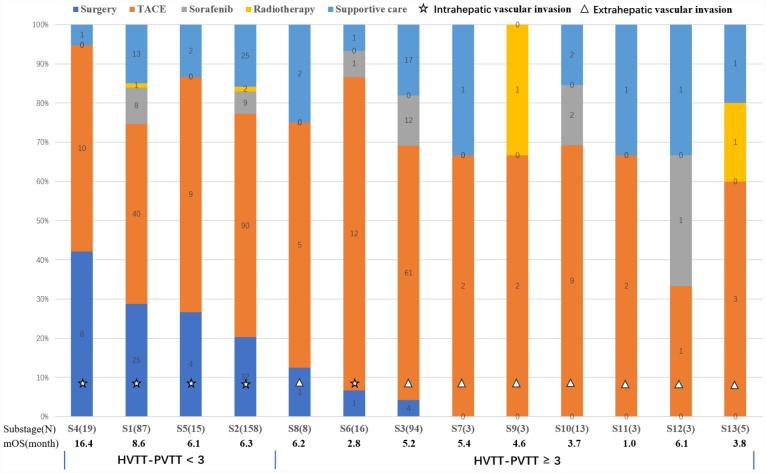
**Graph shows thirteen groups describing different types of vascular invasion including PVTT and HVTT and their proportions of different therapeutic strategies.** The median OS was calculated for each group. The thirteen groups were ranked according to their surgical proportions. Finally, these groups were separated into two groups using the HVTT-PVTT scoring system (< 3 and ≥ 3 points).

The training set was used to build the prognostic classification system. In the training set, patients who had both PVTT and HVTT had significantly poorer outcome than others who had only one type of tumor thrombus (4.63 vs. 6.87 months, *P* = 0.025). We hope to build a new classification including both HVTT and PVTT. Therefore, thirteen groups with different types of vascular invasion were generated (Table 2). To separate patients with different prognosis and surgical proportions, we ranked the thirteen groups according to their surgical proportions and listed their median OS in Figure 1. The top four subgroups had significantly larger surgical proportions and better prognosis than others. In addition, according to the regression coefficients (B) of PVTT and HVTT in the Cox regression model and clinical judgement, different points were given and an equation was built as follows. The HVTT-PVTT score = PVTT (none = 0, segmental branches of portal vein or above = 1, right/left portal vein = 2, and main portal vein or superior mesenteric vein = 3) + HVTT (none = 0, hepatic vein = 1, and inferior vena cava or right atrium = 3). Because inferior vena cava and right atrium tumor thrombus were also extrahepatic vascular invasion like main trunk PVTT, they were also given three points in this equation. We found that the top four groups all had scores < 3 points and other groups had scores ≥ 3 points. Therefore, we classified patients into vascular invasion stage I (HVTT-PVTT < 3 points) and II (HVTT-PVTT ≥ 3 points) with different survival outcomes (7.4 vs 4.6 months, *P* < 0.001) and surgical proportions (24.4% vs 3.6%, *P* < 0.001) by using HVTT-PVTT score. Patients with vascular invasion stage I (HVTT-PVTT < 3 points) are more likely to be candidates for surgery and have better survival outcomes than patients with vascular invasion stage II (HVTT-PVTT ≥ 3 points).

**Table 2:** Vascular invasion sub-classes in the training set.

**Sub-stage**	**Extent of portal vein invasion**	**Extent of hepatic vein invasion**	**Numbers**	**Median OS (month)**	**HVTT-PVTT scores**
S1	I(segmental/sectoral)	None	87	8.6	1
S2	II (left and/or right main trunk)	None	158	6.3	2
S3	III (main trunk)	None	94	5.2	3
S4	None	Hepatic vein	19	16.4	1
S5	I(segmental/sectoral)	Hepatic vein	15	6.1	2
S6	II (left and/or right main trunk)	Hepatic vein	15	2.8	3
S7	III (main trunk)	Hepatic vein	3	5.4	4
S8	None	Inferior vena cava	8	6.2	3
S9	I(segmental/sectoral)	Inferior vena cava	3	4.6	4
S10	II (left and/or right main trunk)	Inferior vena cava	13	3.7	5
S11	III (main trunk)	Inferior vena cava	3	1.0	6
S12	IV (superior mesenteric vein)	Any	3	6.1	3
S13	Any	Right atrium	5	3.8	3

Abbreviations: OS, overall survival; HVTT, hepatic vein tumor thrombus; PVTT, portal vein tumor thrombus.

### Development of a visualized tree-based classification system

**Figure 2:**
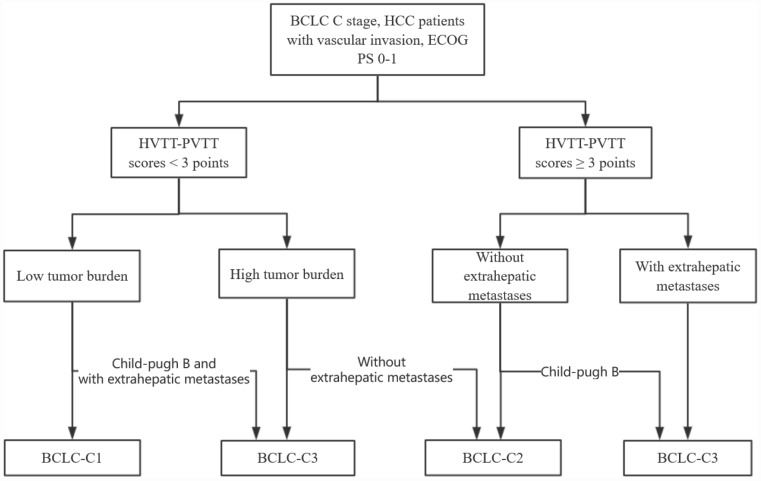
**The final subdivision of HCC patients with macroscopic vascular invasion using classification and regression tree (CART) algorithm.**

Using the Cox regression model, Child-Pugh (stage B vs. stage A) (hazard ratio (HR) with 95% CI=1.383 (1.077-1.777), *P* < 0.05), Tumor burden (high vs. low) (HR with 95% CI= 1.264 (1.005-1.590), *P* < 0.05), Extrahepatic metastases (presence vs. absence) (HR with 95% CI= 1.38 (1.085-1.755), *P* < 0.05), and HVTT-PVTT scores (≥ 3 vs. < 3) (HR with 95% CI= 1.434 (1.130-1.819), *P* < 0.05) were identified as independent prognostic factors of the survival outcome (Table 3). Based on these four prognostic factors and the survival data, the CART algorithm automatically separated patients into subgroups with different prognosis. Subgroups with a similar median OS were merged into larger groups according to clinical judgement to generate the final classification system (Figure 2). Finally, the patients in the training set were classified into three stages. The median OS for these stages was 10.3, 6.1, and 3.3 months, the 1-year survival rates were 48%, 31%, and 18%, and the 3-year survival rates were 22%, 13%, and 9%, respectively (Table 4).

**Table 3:** Univariate and multivariate analyses of OS in the training set.

	**Univariate analysis**			**Multivariate analysis**		
	***P* value**	**Hazard ratio**	**95% confidence interval**	***P* value**	**Hazard ratio**	**95% confidence interval**
Age (>50 vs. ≤50)	0.917	0.988	0.793-1.232			
Gender (Male vs. Female)	0.791	0.947	0.634-1.415			
HBV (positive vs. negative)	0.29	1.302	0.799-2.124			
AFP level (> 400 ng/ml vs. ≤ 400 ng/ml)	0.153	1.116	0.096-1.297			
Child-Pugh (stage B vs. stage A)	0.002	1.474	1.150-1.889	0.011	1.383	1.077-1.777
Tumor burden (high vs. low)	0.002	1.426	1.143-1.778	0.046	1.264	1.005-1.590
HVTT-PVTT scores (≥ 3 vs. < 3)	<0.001	1.578	1.256-1.983	0.003	1.434	1.130-1.819
Extrahepatic metastases (presence vs. absence)	0.001	1.478	1.165-1.874	0.009	1.38	1.085-1.755

Abbreviations: OS, overall survival; HBV, hepatitis B virus; AFP, alpha-fetoprotein;

HVTT, hepatic vein tumor thrombus; PVTT, portal vein tumor thrombus.

**Table 4:** The median OS and 1-, 3-, and 5-year survival rates for training and validation sets.

	**Training set**			**Validation set**		
	**BCLC-C1**	**BCLC-C2**	**BCLC-C3**	**BCLC-C1**	**BCLC-C2**	**BCLC-C3**
Number	148	159	119	164	138	141
Median OS (months, 95% CI)	10.3 (8.02-12.58)	6.1 (4.84-7.36)	3.3 (2.89-3.78)	12.3 (8.84-15.83)	6.9 (5.21-8.58)	5.6 (4.38-6.75)
1-year survival rate (100%)	48	31	18	52	37	28
3-year survival rate (100%)	22	13	9	24	15	5
5-year survival rate (100%)	15	10	0	16	7	2

Abbreviations: BCLC, Barcelona clinical liver cancer; OS, overall survival; 95% CI, 95% confidence intervals.

### Performance assessment

**Figure 3:**
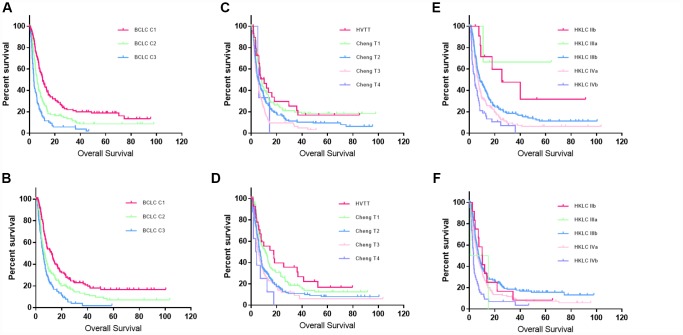
**Kaplan–Meier curves for the training and validation sets.** (**A** and **B**) Kaplan–Meier curves for the training and validation sets created using the new classification system. (**C** and **D**) Kaplan–Meier curves for the training and validation sets created using Cheng’s portal vein staging system. (**E** and **F**) Kaplan-Meier curves for the training and validation sets created using the HKLC system.

After the staging system was built, the validation set was used to assess its performance. The proposed classification scheme could significantly discriminate patients with different prognosis in the validation set (Table 4, Figure 3). The median OS for the different stages was 12.3, 6.9, and 5.6 months, the 1-year survival rates were 52%, 37%, and 28%, and the 3-year survival rates were 24%, 15%, and 5%, respectively. In the training set, the C-index values for this model, Cheng’s portal vein staging system [10], and the Hong Kong Liver Cancer (HKLC) system [17] were 0.63, 0.57, and 0.58, respectively. In the validation set, the C-index values for this model, Cheng’s portal vein staging system, and the HKLC system were 0.60, 0.58, and 0.58, respectively. This prognostic model had better discriminatory ability than the previous portal vein staging system (all *P* values < 0.001) [17].

### Treatment guidance

**Figure 4:**
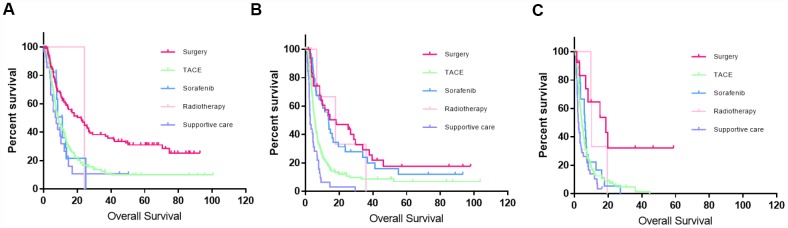
**Kaplan–Meier curves of patients who received different treatment in different stages using this new classification system.** (**A**) Kaplan–Meier curve for BCLC-C1; liver resection significantly improved the survival outcomes compared to those of the other treatments (*P* < 0.001). (**B**) Kaplan–Meier curve for BCLC-C2; although a small proportion of patients still accepted liver resection, the survival benefit was not significant compared to that of those who received sorafenib or radiotherapy (*P* > 0.5). (**C**) Kaplan–Meier curve in BCLC-C3; less than 5% of the patients were eligible for liver resection.

For patients in each subgroup, the first-line treatment strategies included liver resection (48.4%, 10.8%, and 5%), TACE (34.6%, 61.3%, and 67.7%), sorafenib (5.4%, 11.8%, and 19.2%), and supportive care (11.2%, 14.8% and 6.9%). In BCLC-C1, 34.6% (108/312) of the patients received radical surgery; the patients who received liver resection had better survival outcomes than those who received other treatments (median OS: 24.2 months vs 8.5 months; *P* < 0.001) (Figure 4A). In BCLC-C2, the median OS of patients who received radical surgery, sorafenib, radiotherapy, TACE alone, and supportive treatment was 18.3, 13.7, 17.9, 5.6, and 2.8 months, respectively. No significant differences in survival were observed between patients who underwent liver resection and patients who received sorafenib or radiotherapy (*P* > 0.05) (Figure 4B). In BCLC-C3, less than 5% of the patients were eligible for liver resection; the median OS of the patients who received sorafenib, TACE alone, and supportive treatment was 6.1, 4.5, and 2.7 months, respectively (*P* > 0.05) (Figure 4C).

## DISCUSSION

In this study, a HVTT-PVTT score was built to summarize not only the extent of PVTT but also the HVTT that was generally ignored by the previous classification systems. Besides, a new sub-classification system was established by incorporating the HVTT-PVTT score and other critical prognostic factors for personalized management of HCC patients with macroscopic vascular invasion by using the Cox regression model and CART algorithm. The predictive accuracy evaluated by the C-index was better than that of Cheng’s portal vein staging system and HKLC staging system. The new classification could supplement the commonly used BCLC staging system and was meaningful for personalized treatment of HCC patients with tumor thrombus.

In the BCLC staging system, HCC patients with macroscopic vascular invasion are all classified as BCLC-C stage. However, little is known about HVTT due to its rarity. In our article, we noted that among the 869 HCC patients with vascular invasion, 156 patients were combined with HVTT. Kokudo et al. [16] also reported that among 1525 hepatic resections, there were 153 patients who had peripheral hepatic vein tumor thrombus (pHVTT), 21 patients who had major hepatic veins tumor thrombus (mHVTT) and 13 patients who had inferior vena cava tumor thrombus (IVCTT). The median OS in the pHVTT, mHVTT and IVCTT group were 5.27, 3.95 and 1.39 years. Therefore, HVTT should not be ignored when subclassification of HCC with tumor thrombosis was built. In our study, to better classify the extent of vascular invasion of both HVTT and PVTT, thirteen different types of HVTT and PVTT combinations were analyzed. A HVTT-PVTT score was created and finally the HCC patients with vascular invasion were separated into two groups with different survival outcomes (7.4 vs 4.6 months, *P* < 0.001) and surgical proportions (24.4% vs 3.6%, *P* < 0.001).

HCC patients with vascular invasion comprise a heterogeneous population, a subclassification of patients with tumor thrombus is needed to provide better prognostic classification and more suitable treatment guidance for these patients. The HKLC classification showed that more than half of patients with tumor thrombus could benefit from liver resection or TACE over systemic therapy [17]. However, when patients present with intrahepatic tumor thrombus, in the HKLC staging system the prognosis is difficult to predict and the therapeutic strategy seems ambiguous. A subclassification of BCLC-C stage might be more meaningful and useful in clinic. Chung Hwan Jun et al. [22] suggested a new BCLC-C subclassification system for HCC patients based on tumor size, distant metastasis, HCC nodularity, and bile duct invasion, but different types of vascular invasion were not included in the classification system. Sinn et al. [23] subclassified BCLC-C based on portal vein invasion and extrahepatic spread, but other characteristics including hepatic vein invasion, liver function, and tumor status, were not included in their classification. In our study, we not only constructed a new HVTT-PVTT scoring system to classify the extent of vascular invasion of both HVTT and PVTT but also built a comprehensive prognostic staging system using a visualized tree-based classification system. “CART” is a non-parametric decision tree technique that forms a collection of rules based on variables that can dichotomize populations into subjects using suitable parameters [20]. Finally, all patients in the training set were separated into three stages. This new subclassification system performed well in stratifying HCC patients into different prognostic groups in both the training and validation sets. Although the C-index of this classification was relatively low, the biggest advantages of decision tree algorithm were its visualization and user-friendly, which might decrease the predictive accuracy of this classification. In addition, this prognostic model had better discriminatory ability than the previous staging systems for HCC with vascular invasion. In our study, the C-index of Cheng’s portal vein staging system and Hong Kong Liver Cancer system were only 0.57 and 0.58 in the training set.

The corresponding treatment guidance was proposed after analyzing the prognosis of patients in different stages. For patients in BCLC-C1, liver resection significantly improved the prognostic outcomes compared with other treatments. The median OS for patients who received liver resection and other treatments was 24.2 and 8.5 months, respectively. Although a large proportion of the patients received TACE, the median OS was not improved significantly compared with the supportive care group. For patients in BCLC-C2, although many therapeutic strategies could improve the prognostic outcomes compared with supportive care, patients who underwent liver resection or sorafenib had better survival outcomes. However, no statistical difference in the survival outcomes was found between liver resection and sorafenib. For patients in BCLC-C3, although surgery could significantly prolong the median OS, less than 5% of patients were suitable for liver resection. For patients who received sorafenib, TACE alone, and supportive treatment, none of these treatments significantly benefited survival.

This study had several limitations. First, although radiotherapy has been reported to be associated with great survival outcomes for HCC with tumor thrombus, few patients received radiotherapy in this study [24]. Second, only 70 patients (8.05%) in this study received sorafenib therapy. This low proportion might be due to the high costs, date of introduction of sorafenib, and strict medical policy in China. Lastly, the present study was a retrospective and single-center study. External validations and multicenter prospective studies are needed in the future.

In conclusion, our study proposes a comprehensive and visualized tree-based classification system for HCC patients with macroscopic vascular invasion, which may be meaningful and practical for personalized managements of these patients and finally lead to a better survival outcome.

## MATERIALS AND METHODS

### Study design

This study was approved by the institutional review board of the Sun Yat-sen University Cancer Center. Between December 2008 and December 2014, 8599 patients were diagnosed with HCC and treated at Sun Yat-sen University Cancer Center in Guangzhou, including 1228 patients classified into BCLC-C stage. Patients who had no macroscopic vascular invasion or missing medical imaging data, Eastern Cooperative Oncology Group performance status (ECOG PS) ≥ 2, or received treatment previously were excluded. Finally, 869 HCC patients were included into this study. All characteristics were collected, including demographic data, clinical history, baseline laboratory results, Child-Pugh grade, tumor number, tumor size, extent of vascular invasion, extrahepatic metastases, treatment strategies, and survival data.

### Diagnosis

HCC was diagnosed based on AASLD criteria either histologically or through a clinical evaluation based on a typical radiologic appearance in dynamic computed tomography (CT) or magnetic resonance imaging (MRI) and an elevated alpha-fetoprotein (AFP) level [25]. Vascular invasion was defined as a portal vein or hepatic vein tumor thrombus, which was distinguished from a thrombus based on the presence of arterial enhancement and venous expansion or the development of a new thrombus directly contiguous with the tumor. The tumor burden was a composite factor of the size of the largest tumor and the number of nodules in the liver. For simplified definitions of the tumor burden, low tumor burden was defined as a solitary nodule or multiple nodules with no nodule larger than 5 cm, and high tumor burden was defined as multiple nodules with sizes larger than 5 cm [17, 23]. Extrahepatic metastases included lymph nodal or distant organs metastases. Metastases to distant organs was assessed by chest X-ray, bone scan, positron emission tomography (PET)-CT, and chest CT and was diagnosed based on radiographic evidence of a mass in distant organs [26]. Nodal metastasis was defined when fulfilled at least two following criteria: (1) the short axis was larger than 10 mm; (2) in the arterial phase the lymph node was observed contrast-enhanced; (3) the size increased during the follow-up [27, 28]. The medical imaging data from all cases were evaluated by one of the two authors (C.F. and S.L.J.). All patients were followed up until death or loss to follow-up. Overall survival (OS) was defined as the time from diagnosis of HCC to death or the date of last follow-up.

### Subdivision workup of vascular invasion

All cases included in this study were randomly allocated in a 1:1 ratio to build training and validation sets. Then, 426 and 443 patients were allocated into the training and validation sets, respectively. The PVTT were divided according to Cheng’s PVTT classification system into the following categories: type I, tumor thrombus involve the segmental branches of the portal vein or above; type II, the right/left portal vein; type III, the main portal vein; and type IV the superior mesenteric vein [29]. HVTT was categorized into tumor thrombus in the hepatic veins, the inferior vena cava or right atrium. Additionally, for patients who had both HVTT and PVTT. Groups describing different types of vascular invasion were built by considering both the PVTT and HVTT [14, 16, 30]. The surgery proportion and median OS of each groups were calculated. Because many studies have reported that patients who were candidates for surgery had significantly better prognosis than others [13, 14], to identify patients who underwent surgery and also had favorable prognosis, a new HVTT-PVTT score was built according to the regression coefficients (B) of PVTT and HVTT in the Cox regression model and clinical judgement [31].

### Statistical analysis

The characteristics of patients in the training and validation sets were compared. Pearson’s chi-square test and Fisher’s exact test were used to compare categorical variables, and the Mann–Whitney U test was used to compare continuous variables. The Kaplan–Meier method and log-rank test were applied for time-to-event data. Univariate and multivariable analyses were performed using Cox proportional hazards regression. All independent prognostic factors were included in these analyses.

CART algorithm was used to separate the patients into subgroups with similar prognosis using suitable factors. All relevant parameters were imported into the R package “rpart” to automatically separate the patients into different subgroups with different prognosis [32]. Similar subgroups were merged into larger groups manually to simplify and generate the final staging system. The concordance index (C-index) was used to compare the new staging system with Cheng’s and HKLC staging systems, which could both separated these patients into five subgroups [20, 33]. Survival benefits of different treatments in each group were compared using the Kaplan–Meier method and log-rank test [34]. An abstract treatment guideline for the new classification system was proposed. The R (R Core Team, version 3.5.1, Vienna, Austria) and SPSS version 20.0 software (IBM, Armonk, NY) were used for the statistical analysis. *P* values less than 0.05 were considered statistically significant.
